# Successful implementation of a trans-jurisdictional, primary care, anticipatory care planning intervention for older adults at risk of functional decline: interviews with key health professionals

**DOI:** 10.1186/s12913-021-06896-1

**Published:** 2021-08-25

**Authors:** Dagmar Anna S. Corry, Gillian Carter, Frank Doyle, Tom Fahey, Patrick Gillespie, Kieran McGlade, Peter O’Halloran, Nina O’Neill, Emma Wallace, Kevin Brazil

**Affiliations:** 1 Centre for Evidence and Social Innovation, Queen’s University Belfast, Belfast, Northern Ireland, UK; 2 School of Nursing and Midwifery, Queen’s University Belfast, Belfast, Northern Ireland, UK; 3 Department of General Practice, RCSI University of Medicine and Health Sciences, Dublin, Republic of Ireland; 4 Health Economics and Policy Analysis Centre, National University of Ireland, Galway (NUI Galway), Galway, Republic of Ireland; 5 School of Medicine, Dentistry, and Biomedical Sciences, Dunluce Health Centre, Queen’s University Belfast, Belfast, Northern Ireland, UK; 6 Department of Health Psychology, Royal College of Surgeons in Ireland, Dublin, Republic of Ireland

**Keywords:** Anticipatory care planning, Implementation, Healthcare professionals, Primary care intervention, Person-centred, Older adults, Multimorbidity, Trans-jurisdictional, UK, Republic of Ireland

## Abstract

**Background:**

Aging populations present a challenge to health systems internationally, due to the increasing complexity of care for older adults living with functional decline. This study aimed to elicit expert views of key health professionals on effective and sustainable implementation of a nurse-led, person-centred anticipatory care planning (ACP) intervention for older adults at risk of functional decline in a primary care setting.

**Methods:**

We examined the feasibility of an ACP intervention in a trans-jurisdictional feasibility cluster randomized controlled trial consisting of home visits by research nurses who assessed participants’ health, discussed their health goals and devised an anticipatory care plan following consultation with participants’ GPs and adjunct clinical pharmacist. As part of the project, we elicited the views and recommendations of experienced key health professionals working with the target population who were recruited using a ‘snowballing technique’ in cooperation with older people health networks in the Republic of Ireland (ROI) and Northern Ireland (NI), United Kingdom [*n* = 16: 7 ROI, 9 NI]. Following receipt of written information about the intervention and the provision of informed consent, the health professionals were interviewed to determine their expert views on the feasibility of the ACP intervention and recommendations for successful implementation. Data were analyzed using thematic analysis.

**Results:**

The ACP intervention was perceived to be beneficial for most older patients with multimorbidity. Effective and sustainable implementation was said to be facilitated by accurate and timely patient selection, GP buy-in, use of existing structures within health systems, multidisciplinary and integrated working, ACP nurse training, as well as patient health literacy. Barriers emerged as significant work already undertaken, increasing workload, lack of time, funding and resources, fragmented services, and geographical inequalities.

**Conclusions:**

The key health professionals perceived the ACP intervention to be highly beneficial to patients, with significant potential to prevent or avoid functional decline and hospital admissions. They suggested that successful implementation of this primary care based, whole-person approach would involve integrated and multi-disciplinary working, GP buy in, patient health education, and ACP nurse training. The findings have potential implications for a full trial, and patient care and health policy.

**Trial registration:**

Clinicaltrials.gov, ID: NCT03902743. Registered on 4 April 2019.

**Supplementary Information:**

The online version contains supplementary material available at 10.1186/s12913-021-06896-1.

## Introduction

Increasingly, aging populations across the world live with multimorbidity and their complex care poses unprecedented challenges to health systems internationally [1–3], putting a growing strain on systems, healthcare expenditure, staff, and resources [4]. This highlights the importance of building the infrastructure needed to manage aging populations and to apply existing knowledge about chronic disease prevention and treatment in order to find ways to cure or prevent age related diseases and frailty [2, 5]. Health systems are focused on acute care and curative treatments, and are poorly adapted to dealing with patients with multimorbidity who now make up the largest proportion of healthcare utilisation, particularly in terms of primary care services, outpatient visits use and hospital admissions [6–10]. Therefore, transforming health care and working towards a person-centred, primary care-based approach is considered essential with potential significant benefits for patients and health systems alike [7, 11–13]. The objective of such a transformation is a collaborative, integrated care model, designed to provide preventative, personalised care [6–13] at primary care level.

As an integral part of person-centred care, an Anticipatory Care Plan is a summary of ‘ahead’ discussions about the future between the person, those close to them, and the practitioner. It is an active record of the patient’s preferred actions, interventions and responses to inform clinical care, and one that would be developed over time through an evolving conversation, collaborative interactions, and shared decision making. It should be reviewed and updated as medical patient’s conditions or personal circumstances change [14, 15]. It follows that innovative approaches to patient care in the community such as anticipatory care planning (ACP) which seek to facilitate the provision of high-quality, comprehensive and preventive care to older adults at risk of functional decline, require evaluation.

There is a limited evidence base for ACP interventions, and we are hoping to contribute to redressing this scarcity. The current paper constitutes part of the evaluation of a trans-jurisdictional, feasibility randomised controlled trial which tested an ACP intervention in primary care for older adults (≥ 70 years) with multimorbidity and at risk of functional decline [16]. Other parts of the evaluation included interviews with patients; and with implementing stakeholders (i.e., GPs, practice managers, pharmacist, and research nurses); quantitative data including demographics, primary and secondary outcome measures, and health economic analysis. For this paper we elicited the views and recommendations of experienced and established healthcare professionals regarding the viability of the ACP intervention against the background of systemic facilitators and barriers in two jurisdictions: Northern Ireland, United Kingdom (NI) and Republic of Ireland (ROI).

There are two separate health systems on the island of Ireland with significant differences in health policy, structures, coverage, and funding [17]. NI is a region within the United Kingdom that provides an integrated health and social services model of care under the National Health Service which is free to the user at the point of delivery. The ROI has a mixed public-private health care system with all persons resident in the country entitled to receive hospital care through the public funded healthcare system. In addition, the General Medical Services (GMS) card is available to all persons 70 years and over as well as those under 70 who meet a certain income threshold which facilitates the use of the majority of health services free of charge.

## Method

The ACP protocol has been described in detail elsewhere [16]. We interviewed patients about the acceptability of the intervention [18] and implementing stakeholders (i.e., GPs, practice managers, pharmacist, research nurses) about their experience with testing the feasibility of the intervention (under review). The aim of this paper is to report on the expert views of non-participating key health professionals (KHP)s (see Table 2 for occupations) about the benefits and limitations of the intervention, and systemic barriers and facilitators to its’ implementation and sustainability. The key health professionals are individuals who are well placed in the age care field and familiar with this type of service model as reflected by their feedback. They had an advantage point of service planning and policy development, were familiar with regional service planning and policy, and represented the policy shaping community. Each participant group brought very specific insights to provide us with as complete a roadmap as possible for a full RCT.

This paper follows the COREQ guidelines for reporting qualitative research [19] (see Additional file 1) and the TIDieR checklist [20] (see Additional file 2). We used a qualitative descriptive study design [21, 22] and employed conventional content analysis [21, 23, 24] to explore the feasibility of effective and sustainable implementation of the nurse-led ACP intervention for older adults at risk of functional decline. The Consolidated Framework for Advancing Implementation Research (CFIR) [25] guided the evaluation and Table 1 provides an overview of the components of each of Damschroder’s domains to orient the reader to the acronyms used for the respective themes within the findings section.

**Table 1:** Components of the CFIR

**Intervention characteristics (IC)**	Intervention source, evidence strength and quality, relative advantage, adaptability, trialability, complexity, design quality, and cost.
**Outer setting (OS)**	Patient needs and resources, cosmopolitanism (= strength and quality of networks and social capital), peer pressure, external policies, and incentives
**Inner setting (IS)**	Structural characteristics, networks and communications, culture, and implementation climate
**Characteristics of the individuals involved (CIN)**	Knowledge and beliefs about the intervention, self-efficacy, individual stage of change, individual identification with organisation, and other personal attributes
**Process of implementation (PI)**	Planning, engaging, executing, and reflecting & evaluating

We conducted semi-structured interviews with KHPs (see Table 2) to elicit their expert views of the intervention in order to establish its potential viability and sustainability within current health system structures in NI and ROI, and recommendations for successful implementation. The KHPs had no active role in the intervention. They were interviewed via Zoom or telephone according to their preference by researcher DC between January and June 2020, after completion of the intervention.

**Table 2:** Occupation and jurisdiction indicator of participating KHPs

• Voluntary organisation for older people’s health and wellbeing at regional level – managerial	NI 1
• Public Health Agency, Frailty workforce – managerial	NI 2
• Community organisation for older people’s health and wellbeing at local level – managerial	NI 3
• Voluntary sector regional dementia services – managerial	NI 4
• Care Centre manager	NI 5
• Consultant Geriatrician regional Health and Social Care Trust	NI 6
• Speciality Doctor Geriatrics regional Health and Social Care Trust	NI 7
• Care home manager	NI 8
• Consultant Geriatrician at regional Health and Social Care Trust	NI 9
• Independent home share organisation – managerial	ROI 1
• Senior Social Worker, Older Person’s Integrated Care Team	ROI 2
• Speech and Language Therapist	ROI 3
• Public Health Nursing, regional services – managerial	ROI 4
• Occupational Therapist, regional services – managerial	ROI 5
• Speech and Language Therapist – managerial	ROI 6
• Public Health Nurse, researcher and lecturer	ROI 7

Prior to interview the KHPs received a detailed written brief and information on the nature of the ACP intervention, including the program logic model delineating activities, outputs, and objectives. KHPs provided written consent to be interviewed. Interviews were on average 60 min long, were audio recorded with a digital recorder, transcribed verbatim, and were accompanied by field notes.

### Ethical considerations

Ethical approval was obtained in the ROI from the Research Ethics Committee, Irish College of General Practitioners in January 2019 (reference ICGP2018.4.10). In NI, approval was received from the Office for Research Ethics, Northern Ireland (reference 19/NI/0001). All participants provided written, informed consent prior to interview.

### Interview schedule

A semi-structured interview schedule with open-ended questions and prompts, informed by the expertise of the research team [23, 26], was developed consisting of questions pertaining to feasibility of the ACP intervention. Items included questions such as ‘What does Anticipatory Care Planning mean to you?’; ‘What do you think would be the benefits of the ACP intervention in practice?’; ‘What do you think the challenges to supporting/accommodating this intervention in practice would be?’ and ‘How could these be overcome?’; ‘How could we make the process any easier in terms of implementing the intervention in practice?’; ‘Do you see this particular intervention having a role in the future’, - and ‘If yes to above, what would be needed to make this a long-term sustainable intervention?’. Questions were supplemented with open-ended prompts to probe for clarification and depth where appropriate, e.g., ‘What support would be necessary?’, ‘Would changes be necessary for successful implementation?’, ‘What would these changes look like?’. According to preference and owing to the coronavirus pandemic, interviews took place via Zoom and telephone between January and June 2020. The researcher adopted a conversational interview style to establish rapport and ensure participants felt they could talk freely.

### Sample

The 16 KHPs (ROI *n* = 7, NI *n* = 9) purposively selected through snowball sampling via older people health networks in both jurisdictions, with the inclusion criterion of being a health professional working with older people at risk of, or living with, functional decline. We aimed to obtain a multi-disciplinary, maximum variation [23] sample of health experts, evenly distributed across jurisdictions to ensure heterogeneity. We contacted them by telephone to gauge interest and followed up with an email containing information and brief about the study, along with consent forms. 10 of the 26 originally approached KHPs did not engage. Data collection and analysis occurred concurrently and once data saturation was achieved further recruitment was stopped. Table 2 provides a brief overview of which professions were represented, along with their pseudonym denoting jurisdiction.

### The intervention

The goal of the primary-care based, nurse-led and person-centred ACP intervention was to identify unrecognised needs among older (≥ 70years) community-dwelling adults at increased risk of functional decline and to develop a personalised support plan in collaboration with their GP and adjunct pharmacist. A feasibility cluster randomized controlled trial was conducted by assigning eight GP practices to either the intervention or control arm (four per group). Practices were stratified by jurisdiction (NI, ROI) and by rurality prior to randomisation. GP computerised clinical record systems were searched to identify eligible participants and the PRISMA-7 screening tool [27] was used to screen for risk of functional decline (see [16] for full protocol) for inclusion. The inclusion criteria were aged 70+; two or more chronic medical conditions; a PRISMA-7 score of ≥ 3; four or more regularly prescribed medications; a hospital admission in the previous year; three or more physician visits in the past year, and the ability to complete an English language postal questionnaire. All patients meeting the inclusion criteria (*n* = 73) were approached to participate and 65 were recruited. Allocation to the intervention (*n* = 34) or control group (*n* = 29) was conducted on a practice basis.

Registered nurses (RN; *n* = 5) from both jurisdictions completed a three-day training program on their role in the delivery of the ACP intervention. Patients in the intervention group received up to three home visits (up to two hours in duration) by the trained nurses who assessed their physical, mental, and social health with the EASY-Care assessment tool [28], and discussed with them their health goals and plans. Following the RN’s initial visit, assessment, and consultation with the GP, and dependent on the complexity of the identified care or functional needs of the patient, the RN either visited again or contacted the patient by telephone. The holistic ACP assessment, using the EASY-Care tool, was conducted with the aid of a medical summary provided by the GP practice, including details of the patient’s health conditions and prescribed medications. The RN then had a consultation with the participants’ GPs, and an adjunct clinical pharmacist, who conducted a medication review, and through this liaison a personalized care plan was developed and documented.

### Data analysis

The research team managed the data using the software NVivo-12 QSR International. We thematically analysed [21–23] the content of the transcribed interview data and generated an open and modifiable codebook. The CFIR [25] guided the analysis, ensuring that its’ domains (see Table 1) are addressed within the final theme structure. Patterns, commonalities and differences were identified and interpreted, leading to a theme structure and final thematic implementation framework.

To strengthen findings and improve rigour data triangulation (interview data and field notes), source triangulation (a purposive selection of participants from multiple health and social care professions and from two jurisdictions), and researcher triangulation (DC and KB involved in data analysis) were employed, as well as an interview style which allowed participants maximum freedom to speak [26]. Researchers observed reflexivity to minimise potential bias and influence. Pseudonyms (IDs) were used; IDs starting with NI denote participants from Northern Ireland, UK; those starting with ROI denote participants from the Republic of Ireland.

## Results

We thematically analysed content from interviews with 16 KHP from both jurisdictions with the resulting five main themes:

1. Who should receive the intervention;
2. Who should deliver the intervention;
3. The potential benefits of the intervention;
4. What is required for successful implementation; and
5. Systemic barriers.

We have mapped the five major domains of the CFIR [25] (see Table 1) across the main themes as appropriate. Their abbreviations are indicated accordingly in the relevant theme headings below.

### Who should receive the intervention? [IC, OS, IS, CIN, PI]

KHPs suggested the inclusion of vulnerable patients, e.g., mild cognitive impairment (MCI) (NI 1, NI 7, NI 9, ROI 2, ROI 3, ROI 5); mental health problems; complex medical presentations – regardless of age (NI 7, NI 9, ROI 3); cancer patients; palliative care patients; care home residents; those at risk of going into nursing home care; and socially isolated individuals (ROI 2, ROI 5). Involvement of family and care partner was considered important (NI 1, NI 3, NI 5, ROI 1) though not without inherent challenges (NI 7, NI 8, ROI 6).

‘Some people would have less family support, and less knowledge, and are much older - could be living alone; no support around them, so I think it would be very valuable to people like that. People not aware of what’s in their community, what services are available to them.’ (ROI 2).

Given the patients’ right to self-determination, the intervention should be adapted for those who want to retain their autonomy; those who are not coping well with a recent diagnosis; or those who are still very capable and independent (ROI 1, ROI 2, ROI 3). Taking a whole-person approach, and re-engaging with these patients at a later date would be key (ROI 3).

### Who should deliver the intervention? [IC, IS, CIN, PI]

Primary care was seen as the ‘best place’ for the intervention and regarded as very useful to rural GP practices as it could alleviate pressure on GPs who have a geographically wide area to cover. The KHPs suggested that a specially trained nurse in a primary care setting (akin to, and perhaps part of the Public Health Nurses (PHN) in the ROI, and District Nurses (DN) in NI) would be best placed to deliver the ACP intervention on account of the clinical and interpersonal competencies required to fulfill the role (NI 5, NI 6, NI 7, NI 8, NI 9, ROI 2, ROI 4, ROI 6, ROI 7). Their competency framework needs to include the ability to establish rapport with patients, and knowledge of advance care directives and end of life care planning. They should have the ability to negotiate difficult conversations with sensitivity and empathy, applying a person-centred, individualistic approach, and making patients feel comfortable and validated. See Table 3 for KHPs views on the role, setting, and aspects of the nurse-led ACP intervention.

**Table 3:** KHPs views on the role, setting, and aspects of the nurse-led ACP intervention

**What do ACP nurses / practitioners do?**	• Working with GPs for patient selection according to eligibility criteria • Regular home visits (needs-based duration) • Identify risk of functional decline • Holistic assessment • Active listening • Person-centred care • Co-developing and regular reviewing of advance care plan with patient (and family carer / advocate) • Work with pharmacist for medication review • Liaise with geriatricians, physios, OTs and other health professionals • Liaising with, and signposting to, the voluntary and community sector • Have a level of seniority
**Where are they located?**	• Based in primary care • Within Trusts (NI) and Community Health Organizations (ROI) • Allied to Older People Care, and Geriatricians
**What structures and support are required for this service?**	• Inclusive eligibility criteria with right to self-determination • ACP training incorporated into UG student curriculum • Ongoing ACP training available for qualified nurses • Supervision for ACP nurses • Shared electronic records • Standardized procedures • Competency framework • Structured approach • Integration in to existing structures in both jurisdictions for this patient group (avoiding duplication) • Implementation in line with current health system reforms in both jurisdictions

‘I am conscious of the need to be reviewing medications and clinical diagnosis and that. So, nursing probably would be best placed, to be fair.’ (ROI 2).

### The potential benefits of the intervention [IC, CIN, PI]

The potential for significant long-term benefits of the intervention were recognized by the KHPs ‘I mean, it is vital.’ (ROI 1), so much so that they regarded it as essential for older person care. Facilitated by a structured care plan, quality of life could be improved through a comprehensive holistic assessment, reduction of polypharmacy, and prevention of further decline with associated crisis intervention (NI 1, NI 6, ROI 1, ROI 6). The potential of the intervention to reduce social isolation was discussed (NI 1, ROI 1, ROI 2).

‘It’s a very crucial intervention, I think; that here is a policy to try and have alternatives to acute admissions, and to have alternatives to care in the hospital setting is very important for older people.’ (NI 6).

‘We could really enhance the quality of people’s lives. I actually believe we could take the pressure off the hospital system. And it would save money.’ (ROI 3).

It was recognized that the ACP intervention puts patients in control and allows them to co-design their health care and help alleviate any anxiety about their future trajectory (ROI 6).

### What is required for successful implementation? [IC, OS, IS, CIN, PI]

The main theme of ‘what is required for successful implementation’ included the subthemes ‘what is needed at systemic level’ (with the sub-theme ‘maximise existing structures and avoid duplication)’and ‘what is needed at intervention level’ (with the sub-themes: ‘patient assessment tool’, ‘home visits are highly suitable’, and ‘medication review is very useful’).

#### What is needed at systemic level

KHPs indicated that multi-disciplinary, integrated working would be essential for the success of this intervention (e.g., NI 4, NI 6, NI 9, ROI 6, ROI 7). They felt that policy changes, including an identified pathway, may be required to accommodate this, along with standardization (e.g., NI 1, ROI 6) across regions and an awareness of the possibility of creating inequality due to GPs in ROI not being obliged to buy-in. Specific, person-centred training for the nurses, ideally incorporated in their undergraduate training, along with access to peer support and good supervision (e.g., NI 3, NI 5, NI 6), were seen as essential, with education and health systems working in tandem: ‘Bring us all into the same room’ (ROI 5). Equally, GPs and other health and social care professionals who are involved in the intervention should be part of the training too.

Changing public perception and increasing awareness and understanding of the nature of ACP and its’ preventative role in functional decline was regarded as essential (NI 4, NI 5, NI 6, NI 7, NI 9).

#### Maximise existing structures and avoid duplication

While concerns were voiced that aspects of the ACP intervention may duplicate efforts that are already in place (NI 3, NI 4, NI 6, ROI 1, ROI 7) it was noted that these are not community based, and lack structure. Duplication is always a potential risk in service provision, which is important to acknowledge, however, KHPs recognized the importance of this intervention and its integration with existing services. The community and voluntary sector were said to be an essential facilitator for the ACP service (NI 1, NI 3, NI 4, ROI 2). The importance of establishing links for potential collaboration with relevant bodies was highlighted (ROI 2, NI 3, NI 1).

#### What is needed at intervention level

KHPs provided a very clear directive in terms of what structures and procedures would need to be in place to facilitate the ACP intervention. As this is a primary care intervention it is key to have GPs support (NI 1, NI 2, NI 4, NI 5, NI 7, ROI 4). Equally important is the collaboration with other healthcare professionals, including a pharmacist to carry out the medication review. Ideally, medical records would be electronic and shared by all health professionals involved while adhering to data protection requirements (NI 3, NI 4, NI 6, NI 7, ROI 4, ROI 6, ROI 7) to ensure timely and accurate communication and data sharing in the interest of best practice and care. As yet, fully shared electronic records are not commonplace in either jurisdiction.

‘It’s so hard when we are all paper-based to even work out what’s happening.’ (ROI 7).

Adherence to a protocol and a known intervention pathway should ensure equality of access. Timely and accurate screening is essential to ensure best outcomes, and needs to follow the same protocol across all GP practices. Education about safety procedures (e.g., in the event of a fire) ought to form part of the ACP intervention (NI 9).

#### Patient assessment tool

KHPs indicated that the patient assessment tool should include a quality-of-life measure (NI 2), account for literacy limitations (ROI 2), contain questions pertaining to present and future communication ability, as well as asking ‘What are your circumstances and / or conditions stopping or limiting you from doing?’. It was emphasised that the assessment and interpersonal skills of the person administering the questionnaire are almost more important than the tool itself, therefore, suitable training and expertise are vital.

‘It’s the person doing the assessment who actually has eyes and ears themselves and knows the person inside out, and knows their environment that they’re living in. That’s the only way you can make a really good assessment.’ (ROI 7).

#### Home visits are highly suitable

Home visits were regarded as the appropriate approach for the ACP intervention (ROI 3, NI 3, NI 6, NI 7, NI 8, NI 9). The reasons were four-fold: (1) It is a lot easier to obtain a full and true picture of the patient’s circumstances and functioning in their own environment, along with supports available to them; (2) It ensures that all eligible patients are reached. Often patients who depend on their family or others to take them to and from appointments, are loath to ask for help as they do not want to burden them; (3) Patients are more comfortable in their own environment and, therefore, more inclined to have open discussions; and (4) It eases the workload for GPs, particularly in rural areas, in looking after patients who are not mobile.

‘Having an assessment at their own homes is a very good idea. I fully second that and having a specialist nurse for that.’ (NI 7).

#### Medication review is very useful

The medication review was regarded as ‘hugely beneficial’ to help reduce potentially harmful polypharmacy (ROI 2, ROI 4, ROI 7, NI 6, NI 8, NI 9). The ACP nurse working closely together with the GP and pharmacist to facilitate medicine optimization (ROI 4) would help alleviate pressure on PHNs (ROI 7) who are currently undertaking this task as part of their remit.

‘There’s a real need for a medication review and, you know, it will be a rarer person that wouldn’t have medications adjusted or, you know, moved in some way.’ (NI 6).

The fact that the ACP nurse would have direct access to out-of-date medications as part of their home visit, and would be able to provide a much more efficient review was seen as something that would be very beneficial to patients and GPs alike (ROI 7).

### Systemic barriers [OS, IS]

Systemic barriers to successful implementation were identified by KHPs in both jurisdictions with some overlap as detailed in the following sections. These barriers speak to the intervention characteristics, inner, and outer settings domains of the CFIR [21], the latter two of which differ somewhat across the jurisdictions.

#### National Health Service, United Kingdom (NHS)

In NI, geographical differences in access to health services were seen as a barrier to successful implementation (e.g., NI 1, NI 6, NI 8). Health Trusts have different services and funding which could be detrimental to an equitable ACP service across NI, and presents a barrier to the sustainability of the service model. The service inequality across Trusts has been likened to a ‘postcode lottery’ (NI 1).

‘Really and truly, there’s lots of areas of deprivation in Northern Ireland with very few services in.’ (NI 1).

KHPs felt that the ACP intervention would require a cultural change: a move from the medical model (treating a condition) to a person-centred, biopsychosocial model (treating the whole person) of care, with the focus on prevention rather than treatment (e.g., NI 1, NI2, NI3).

‘I suppose one of the main challenges around multiple comorbidities is the number of people who interface and number of professionals who interface with older people, and how confusing that is for older people. We broke the body into systems and organs, and sometimes people don’t feel like they’re being treated as a person. They’re being treated as a condition, and I think that’s really challenging.’ (NI 2).

Lack of time (NI 2, NI 3, NI 3, NI 5, NI 7, NI 8, NI 9), funding (NI1, NI 2, NI3, NI 4, MI 7), staffing and resources (NI 5) were identified as main barriers, particularly in light of an aging population with multimorbidity requiring long-term, complex care. It was pointed out that systemic changes must be accompanied by educational changes (NI 1, NI 2, NI3, NI5, NI 6) so that relevant health professionals have the required skills and competencies for the ACP intervention. Insufficient integrated working, reflected in the absence of universally accessible, shared medical records (NI 4, NI 6, NI 7, NI 9), would make the ACP intervention challenging (NI 4).

#### Health Service Executive, Republic of Ireland (HSE)

In the ROI, a sizeable barrier was identified as service fragmentation as GPs and public health nurses (PHNs), despite being under the umbrella of Primary Care Teams, work separately and independently - ‘in silos’, and hold separate patient records (‘We don’t have universal medical records in the South.’ (ROI 6)), and separate care plans, leading to poorly integrated services (ROI 2, ROI 4, ROI 5, ROI 6) and a lack of efficiency. GPs support of the ACP program was considered essential.

‘Basically, it means that a GP might have one plan, the PHN might have a totally different plan. And the OTs and physios might not be aware of any plan.’ (ROI 4).

The system was seen as still largely adhering to the medical model (ROI 3, NI 2, NI 5), and focused on individual conditions (e.g., ROI 3) rather than the whole person. Long waiting lists for multidisciplinary primary care teams make a timely care plan difficult. Patients are aware that the system is overstretched which can cause a lot of anxiety.

 Regional inequalities in terms of access and service provision were discussed, and the role the mixed private-public health system in ROI plays in these disparities (ROI 4, ROI 7). The HSE GMS card presents a barrier (ROI 2, ROI 4) as it is means-tested, and not everybody has one, i.e., 53 % of over 65 s and 20 % of over 70 s [29] are without a GMS card, and therefore are not entitled to a PHN visit or additional community supports free of charge.

#### Shared systemic problems

A lack of funding, capacity, and resources (e.g., NI 2, NI 5, NI 9, ROI 1, ROI 3, ROI 6) was identified by KHPs in both jurisdictions, as were regional inequalities, and a lack of integrated working. The scaling up of the intervention to the full, eligible patient population was considered challenging against this background. The lack of integrated working, particularly with GPs, was heightened in the ROI, and the lack of universally shared electronic records was pointed out in both jurisdictions. In terms of CFIR [22] domains, results showed shared challenges yet structural differences in health systems across jurisdictions (IS), which would impact on the process of implementation (PI).

## Discussion

Essential clinical and interpersonal competencies of the role of ACP nurse have been identified as the ability to conduct a holistic assessment, triage, health and social care systems navigation, a level of seniority, and receiving ACP specific training; with key skills and attributes to include active listening, empathy and compassion (Theme 1). GPs were thought to be best placed to provide anchorage for this partnership care model, although concerns have been voiced as to time requirements. GPs could play a significant role in the future delivery of care to those living with chronic conditions, but as part of a model in which the responsibility was shared across multidisciplinary teams and settings [29] (Theme 2).

Potential benefits of the ACP intervention were said to include the medication review which would optimise prescribing, reduce cost and improve health [30]. Home visits, health education, a tailored regimen, and rapport building have the potential to increase adherence rates [31], while loneliness and isolation could be diminished. Patients stand to gain a greater understanding of both their future care needs and of ways in which they can prevent further functional decline and improve their quality of life (Theme 3].

There was consensus among the KHPs both in NI and in ROI on sufficient funding, staff, time, and resources being central requirements to the success of the intervention. Additionally, specific actions were identified as necessary for a successful and sustainable ACP intervention, e.g., widening inclusion criteria (age, MCI); maximising existing structures and collaborating with established services within both health systems, thereby avoiding duplication; linkages with the voluntary and community sector where possible, multidisciplinary, relevant policy changes and standardisation, an identified pathway, GP / GP Federations (NI) and CHO (ROI) anchorage respectively, with support from Geriatrics; increasing public health literacy, perception and understanding of ACP; appropriate training as part of the nursing undergraduate programme; peer support and good supervision. As suggested by KHPs, choosing the most appropriate assessment tool for the intervention going forward is paramount so that duplication is avoided while objectives are achieved. This theme provides some essential building blocks for the ACP intervention in both jurisdictions (Theme 4).

Differences and commonalities in health services, both at systems level as well as at grass root level, need to be considered when upscaling the intervention. Systemic barriers need to be circumnavigated skilfully, and facilitators optimised and utilised to ensure effective and sustainable implementation of the ACP service at regional level. In particular, differences in access arrangements, and in reimbursement process, across the healthcare systems in NI and ROI, may require variations in the approach adopted to engage both healthcare professionals and patients to facilitate the successful implementation of the intervention. Structural differences between the HSE and the NHS (IS) were highlighted, as were the differences in health policies and networks across jurisdictions (OS), both of which affect the process of implementation (PI). This highlights the importance of taking a context specific approach to implementation (PI), adapting the ACP intervention (IC) to jurisdictional and regional settings and characteristics (Theme 5).

### Limitations

A snowballing sampling technique may carry the potential of bias which could be limiting; and KHPs were not directly involved in the feasibility trial of the intervention, but based their views and recommendations on the written information and brief supplied to them, against the background of their own professional expertise and experience. The study focuses on the feasibility of the ACP intervention and the intervention still requires formal evaluation via a full-scale RCT. The strengths of the study include source, data, and researcher triangulation to strengthen findings and increase rigour; one researcher conducting all interviews, ensuring consistency and validity; and converging findings from two jurisdictions, increasing validity.

## Conclusions

Notwithstanding independent and structurally different health systems in NI and ROI, older people at risk of functional decline share the same health and social care needs across the jurisdictions. The data obtained from KHPs in both health systems indicate that, with some adjustments to, and flexibility in, the process of implementation in response to jurisdictional differences in health system structure, the primary care based, nurse-led ACP intervention would not only be a very valuable tool in both meeting current patient needs and alleviating pressure on secondary care, but also be a highly appropriate and timely service in light of the long-term reform plans [32, 33]. Potential benefits of this preventative service model include better quality of life and fewer hospital and care home admissions. While the study was conducted on the island of Ireland it is trans-jurisdictional and thus highlights the international application of the findings.

## Supplementary Information

**Additional file 1.** Consolidated criteria for reporting qualitative studies (COREQ): 32-item checklist.

**Additional file 2.** The TIDieR (Template for Intervention Description and Replication) Checklist

## Data Availability

The data underlying this study cannot be shared publicly because they are qualitative patient interviews which contain personal and potentially identifiable information, and participants have consented to publication of anonymous quotes only. Requests for data can be made to Queen’s University Belfast Research Governance, Ethics and Integrity department (researchgovernance@qub.ac.uk) with appropriate ethical approval.

## Abbreviations

ACP: Anticipatory Care Plan
ANP: Advanced Nurse Practitioner
CFIR: Consolidated Framework for Advancing Implementation Research
CIN: Characteristics of the Individual Involved
DN: District Nurse
GMS: General Medical Service
GP: General Practitioner
HSE: Health Service Executive
IC: Intervention Characteristics
IS: Inner Setting
KHPs: Key Health Professionals
MCI: Mild Cognitive Impairment
NHS: National Health Service
NI: Northern Ireland
OS: Outer Setting
PHN: Public Health Nurse
PI: Process of Implementation
RN: Research Nurse
ROI: Republic of Ireland
WHO: World Health Organisation
